# The Interaction of Canine Plasminogen with *Streptococcus pyogenes* Enolase: They Bind to One Another but What Is the Nature of the Structures Involved?

**DOI:** 10.1371/journal.pone.0028481

**Published:** 2011-12-09

**Authors:** M. Judith Kornblatt, Jack A. Kornblatt, Mark A. Hancock

**Affiliations:** 1 Department of Biology, Concordia University, Montreal, Quebec, Canada; 2 Department of Chemistry and Biochemistry, Concordia University, Montreal, Quebec, Canada; 3 Centre for Structural and Functional Genomics, Concordia University, Montreal, Quebec, Canada; 4 Sheldon Biotechnology Centre, McGill University, Montreal, Quebec, Canada; Semmelweis University, Hungary

## Abstract

For years it has been clear that plasminogen from different sources and enolase from different sources interact strongly. What is less clear is the nature of the structures required for them to interact. This work examines the interaction between canine plasminogen (dPgn) and *Streptococcus pyogenes* enolase (Str enolase) using analytical ultracentrifugation (AUC), surface plasmon resonance (SPR), fluorescence polarization, dynamic light scattering (DLS), isothermal titration calorimetry (ITC), and simple pull-down reactions. Overall, our data indicate that a non-native structure of the octameric Str enolase (monomers or multimers) is an important determinant of its surface-mediated interaction with host plasminogen. Interestingly, a non-native structure of plasminogen is capable of interacting with native enolase. As far as we can tell, the native structures resist forming stable mixed complexes.

## Introduction

The idea that there are components on the cell surface that bind plasminogen and result in its activation to plasmin was put forward by Miles and Plow in 1985 [1]. The cell surfaces in question were mammalian and the original observation dealt with platelets. It was clear from the outset that there was more than one receptor on the cell surface and the identity of these receptors was soon established [2]. Amongst the mammalian cell surface molecules that can potentially bind plasminogen are the gangliosides [3] as well as α-enolase [4]–[10] and glyceraldehyde-3-phosphate dehydrogenase [11].

Pathogenic bacteria also have mechanisms for activating plasminogen, such as the secretion of streptokinase or staphlokinase [12], [13]. In addition, some pathogenic bacteria and eukaryotic parasites have cell surface molecules that can bind plasminogen; the binding, in this case, might serve an insidious role. If binding by a cell surface molecule on a pathogen can result in activation of plasminogen to plasmin, then the binding-activation event might help disseminate the infection [14], [15]. For many of these bacteria, enolase has been identified as a cell surface plasminogen receptor [13], [15]–[41]; the binding of plasminogen and similar proteins to enolases from *Streptococci* has been studied in detail [13], [24], [33], [36], [42]–[45].

Plasminogen is universally present in the blood of mammals. In solution it is a monomeric protein composed of an N-terminal peptide, followed by five kringle domains, followed by a preproteolytic domain [46]. The N-terminal domain is non-covalently associated with kringle 5 to form a structure which doubles back on itself like a string of beads. There are partial X-ray or NMR structures of the components of plasminogen but no complete structure.

**Figure 1:**
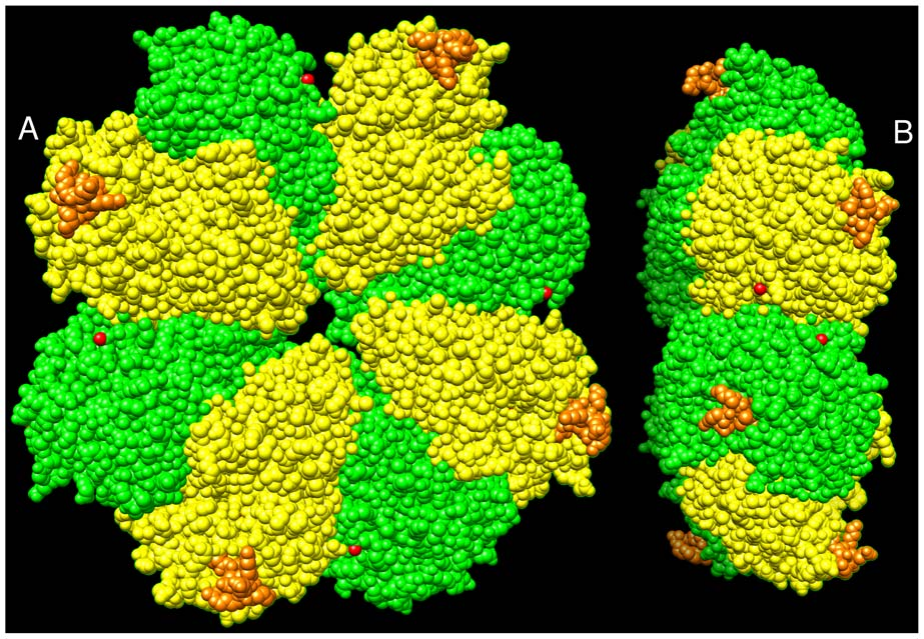
Structure of the octameric enolase. The identical subunits are arranged as a tetramer of A-B pairs. The color coding is: “A” subunits are green and “B” subunits are yellow. The two putative plasminogen binding sites are shown. The first: only one atom of the C-terminal lysine-433 is visible in the X-ray structure. It is coloured red. The adjacent leucine-432 is also coloured red for convenience of viewing. The second: the putative site consists of residues 248–256. It is coloured orange. Figure 1A. Top down view showing the positions of the two sites. On the A subunits, the second site is easily seen; it shows up on the B subunits when the molecule is flipped through 180°. Figure 1B. An end on view of the octamer. The orange second site is clearly exposed whereas the red C-terminal is mostly buried.

In contrast we know significantly more about the structure of the enolase used here. Enolases from *Streptococci* are homo-octamers in which the monomer consists of about 435 amino acids. The eight identical subunits are arranged as a tetramer of dimers. The crystal structure of the enolase from *S. pneumoniae* is shown in Figure 1 (1W6T.pdb) [44]. Based on small molecule studies, each monomer contains two potential binding sites for plasminogen (Fig. 1). One site consists of the two C-terminal lysines, coloured red in Figure 1; the second, coloured orange, consisting of residues 248–256, is on an exposed loop (Figure 1). The sequence of this loop, including the presence of two lysines, seems to vary among the *Streptococci* [13].

All the work reported here has been done on the octameric protein from *S. pyogenes*. Lysines 252 and 255, on an internal loop, and the C-terminal lysines, 434 and 435, are required for binding [47]. A homology model, based on the *S. pneumoniae* structure is available [47].

We have worked with native, solution phase, canine plasminogen (dPgn) and native, solution phase, enolase from *Streptococcus pyogenes* (Str enolase) and have not been able to demonstrate that one protein will bind to the other. What is there that is different about our studies that distinguishes them from the others? We have been able to demonstrate binding when one of the proteins is not in a soluble, native conformation. In this report we establish under what conditions binding will occur and we establish some of the characteristics of the species that are involved.

## Results

Four forms of native Str enolase were used in this study. Unless otherwise stated, experiments were performed using the doubly mutated form of Str enolase, F137L/E363G; this protein contained an N-terminal hexa-histidine-tag. The three other variants used were: (1) wild type enolase with no tag, (2) wild type enolase with an N-terminal hexa-histidine tag, (3) wild type enolase with C-terminal deca-histidine tag. Following purification, all four were active, correctly folded according to their circular dichroism spectra (not shown), and at least 90% octameric (as determined by sedimentation velocity in the analytical ultracentrifuge, data not shown). We refer to protein with these characteristics as “native”.

Most experiments used canine plasminogen in its native closed form.

We have applied the following techniques to determine when and under what conditions Str enolase will bind to plasminogen: fluorescence, isothermal titration calorimetry (ITC), dynamic light scattering (DLS), analytical ultracentrifugation (AUC), pull down reactions, and surface plasmon resonance (SPR). The critical information that must be kept in mind is that the two proteins bind under some circumstances and do not bind under others. Understanding the difference between the two sets of conditions is the thrust of this work.

### Solution methods with native proteins

**Figure 2:**
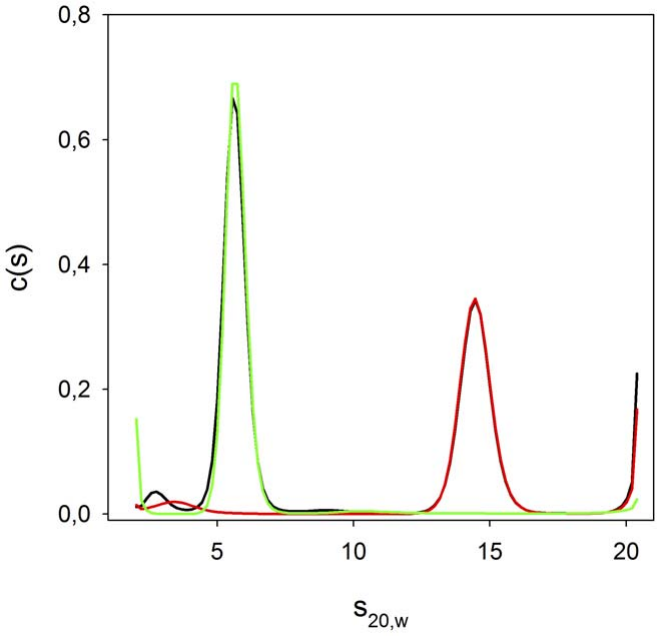
Analytical ultracentrifugation of Str enolase, dPgn, and a mixture containing a ratio of 1 Str octamer∶1 dPgn. The solid black line is the combined Str enolase, dPgn mixture. The red line is the Str enolase, and the green line is the dPgn. There is almost perfect overlap between the red line and the Str enolase portion of the mix. There is almost perfect overlap between the green line and the dPgn portion of the mix. All proteins were ca 0.010 mM. The centrifuge speed was 32, 000, 20°C. The data were analyzed using Sedfit.

Three different techniques were used in which we could not demonstrate an interaction between the two proteins: (1) dynamic light scattering, (2) sedimentation velocity with the analytical ultracentrifuge, and (3) fluorescence polarization. The experiments were performed in buffers at pHs between 6.5 and 7.4 where we knew the Str enolase was mainly octameric and the plasminogen was in its native state. Various concentrations, up to 0.010 mM, of the two proteins and various ratios were used; in no case was there any evidence of an interaction. The results of the AUC experiment are shown in Figure 2. There are no significant differences between the sedimentation behavior of the individual proteins and their sedimentation behavior in the mixture. The S_20, w_ values are precise to about 0.5%. In this case, there is no question but that the two proteins do not interact on the time scale of the experiment. The results of a DLS experiment are shown in Table 1. DLS will not resolve differences between proteins whose size differs by less than a factor of two. Accordingly, in the mixture of dPgn and Str enolase the data resemble those of the Str enolase. The significant aspect of the data in Table 1 is that there is not a larger species that has formed on incubation of the two individual native proteins.

**Table 1:** Stokes' Radii of Str enolase, dPgn and their mixture.

Sample	major species, hydrodynamic radius
dPgn	4.2 nm99.9% mass
Str enolase	6.7 nm99.9% mass
Mix 8∶1dPgn∶octamer	6.2 nm99.9% mass

The protein radii were determined by dynamic light scattering using a Wyatt, DynaPro apparatus thermostated at 20°C. Protein concentrations were kept at levels which would give a maximum of 2E6 counts per second but always more than 3E5 counts per second. The acquisition time was 10 seconds and 100 acquistions were collected. At least 95 of the 100 acquisitions were acceptable in each evaluation.

### Solution methods with non-native proteins

The above experiments were performed with dog plasminogen; previous studies (see references in Introduction) used human plasminogen. Could this be the reason for our failure to observe binding? A fluorescently labeled, catalytically inactive, derivative of human plasminogen (5′IAF-Pg S741C) was tested for its ability to bind to Str enolase; this protein was the generous gift of Dr M.E. Nesheim (Dept. of Biochemistry, Queen's University, Kingston, Ontario. Dr Nesheim died on June 4, 2011). The fluorescent label was used to monitor the diffusion coefficient of plasminogen; binding to enolase would result in a decreased diffusion coefficient, due to the increase in size and, hence, an increase in polarization. The results are shown in Table 2. The first two lines of the table show that there is no detectable change in polarization when the proteins have been maintained in their native state at pH 6.7. As can be seen in Table 2, the polarization of the human plasminogen was only changed when we lowered the pH of the enolase solution to 4.5 and then diluted the enolase into phosphate buffer, pH 6.7. At the submicromolar concentrations of Str enolase used in this experiment, there was no problem with the protein precipitating. Our reasons for performing this pH change will soon be apparent. Under these conditions, there was a significant increase in polarization of the human plasminogen, indicating that binding of enolase had occurred. We concluded that our failure to observe binding in the earlier experiments was not due to using dog plasminogen; we also concluded that binding involved non-native form(s) of Str enolase. It is interesting to note that Antikainen et al. [48] observed that enolase bound to the cell wall of *Lactobacillus crispatus* at pH 5 but not at higher pH. This binding may also involve a non-native form of enolase that forms at low pH.

**Table 2:** Fluorescence polarization of Str enolase interacting with IAF-Pg S741C-hPgn.

Potential Ligand of IAF-Pg S741C-hPgn	Treated first at pH and measured at pH 6.7	P (polarization)
None	6.7	0.30±0.01
Str enolase	6.7	0.30±0.01
Str enolase	4.5	0.43±0.01

Fluorescently-labeled hPgn was used at a final concentration of 140 nM. Str enolase, when present, was 0.057 mM. The temperature was kept at 20°C. The Eclipse fluorometer settings were: slits, 5 nm excitation and emission wavelengths were 470 nm and 516 nm respectively. The G factor was determined for each measurement. All measurements were done five times; the spread between the calculated polarization values was never more than ±0.01. The entire experiment was repeated one time.

**Figure 3:**
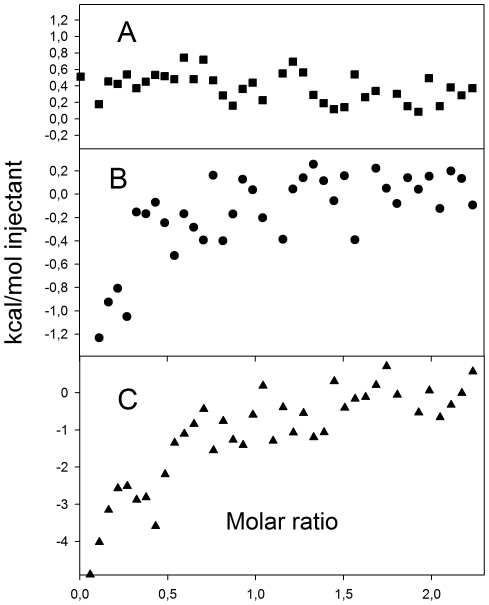
Binding of plasminogen and enolase determined by ITC. (A.) Native Str enolase was titrated into native dPgn. There was no detectable binding. (B.) Str enolase was brought to pH 9. It precipitated and was dialyzed to bring the pH back to pH 6.9. The protein loss was about 50%. The remaining protein retained 30% enolase activity. Enolase was titrated into dPgn. There were 0.18 sites available for binding on the average enolase monomer. (C.) Str enolase was brought to pH 4.5. It precipitated. The precipitate was dialyzed back to pH 6.9 and then dissolved in 27% urea (final) and then dialyzed again at pH 6.9. Approximately 30% of the original enolase was recovered and that retained about 30% of its specific activity. Enolase was titrated with dPgn. This protein bound 0.34 dPgn per average monomer of Str enolase.

Isothermal titration calorimetry (ITC) was also performed using both native Str enolase and enolase treated at pH 9.0 (chosen to mimic conditions of ELISA assays) or pH 4.5 (chosen to mimic some aspects of SPR assays). The results are shown in Figure 3. When both dPgn and Str enolase are maintained at pH 6.9, there is no detectable binding (Figure 3A). When the enolase has been incubated at pH 9.0 and then slowly returned to 6.9 (Figure 3B) or treated at pH 4.5 and then returned to pH 6.9 (Figure 3C) binding occurs. At the high concentrations required for the ITC experiment, enolase treated at either pH 9.0 or 4.5 precipitated. Following dialysis at pH 6.9, about 50% of the protein that had been treated at pH 9.0 was recovered. Enolase treated at pH 4.5 remained insoluble after return to pH 6.9. It was therefore solubilized in 27% (wt/wt) urea and dialyzed again at pH 6.9 before being used for the ITC experiment. 30% of the original protein was recovered. In both cases, the specific activity of the final enolase preparation used in the ITC experiments was only 30% of that of the original, untreated enzyme. The data are clear and are consistent with the results of the fluorescence polarization measurements. Binding does not occur when enolase is in its native conformation. When enolase has been subjected to a treatment that alters its conformation, and its enzymatic activity, as in going from a native to a denatured state, there is the potential for binding.

**Figure 4:**
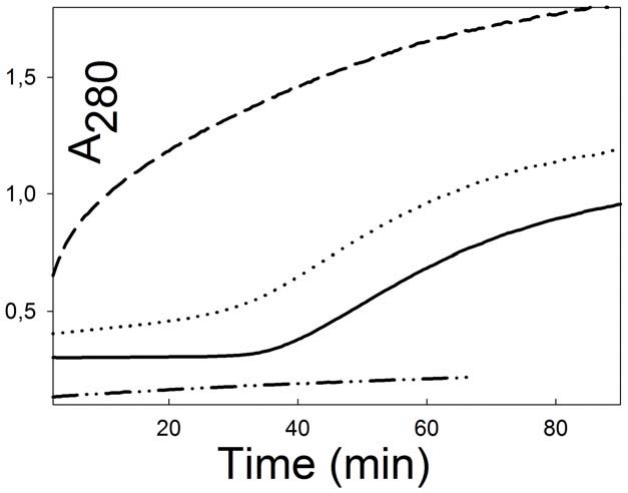
The influence of Str enolase on the precipitation kinetics of dPgn. Each sample contained 0.2 M DTT. The bottom trace (dash dot) contained 0.019 mM Str enolase but no dPgn. The solid trace contained 0.002 mM dPgn but no Str enolase. The dotted trace contained 0.002 mM dPgn and 0.0019 mM Str enolase. The top trace (dash) contained 0.002 mM dPgn and 0.019 mM Str enolase. Under the reducing conditions shown here, Str enolase enhances the rate at which dPgn precipitates. In the absence of reductant, there is no precipitation of the two proteins over a period of days.

**Figure 5:**
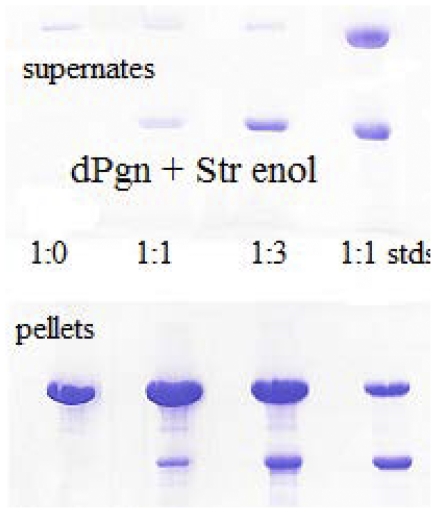
Str enolase and dPgn co-precipitate in the presence of reductant. SDS-PAGE of pellets and supernates from an experiment similar to that described in Figure 4. The mixtures containing the proteins and the reductant were centrifuged and the phases separated. The upper and lower lanes 4 contain standards; 1∶1 dPgn and Str enolase (monomers); the top band is the 90 kDa dPgn and the bottom is the 45 kDa Str enolase monomer. The lanes 1 show that dPgn precipitates almost quantitatively in the presence of reductant. Lanes 2 shows that the precipitating dPgn pulls down enolase from a 1∶1 mix of dPgn and Str enolase monomer but the precipitation is not quantitative. Lanes 3 show that the mixture which contains a 1∶3 ratio of dPgn to Str enolase monomer will pull down a significant fraction of the Str enolase.

There is one condition to date in which we have seen binding between native Str enolase, in solution, and dPgn. If dPgn is treated with reductant, such as dithiothreitol (DTT), the dPgn partially opens (as if it were a molten globule) and precipitates [49]. The precipitation can be monitored by light scattering at 280 nm. Figure 4 shows the time course of precipitation of dPgn by itself and in the presence of Str enolase. Str enolase, by itself, does not precipitate; however, its presence increases the rate at which dPgn precipitates. The samples from a similar experiment were centrifuged and both the supernates and pellets subjected to SDS-PAGE. As can be seen in Figure 5, the precipitates contain both dPgn and Str enolase. When the ratio of dPgn to Str enolase was 1∶1, 12% of the precipitated protein was Str enolase, corresponding to a dPgn∶enolase ratio in the pellet of 6∶1. When the ratio in the initial incubation was 1∶3, 25% of the precipitated protein was Str enolase, giving a 3∶1 ratio. A precipitating form of dPgn binds native Str enolase that is in solution.

### Binding between plasminogen and Str enolase in the presence of a surface

**Figure 6:**
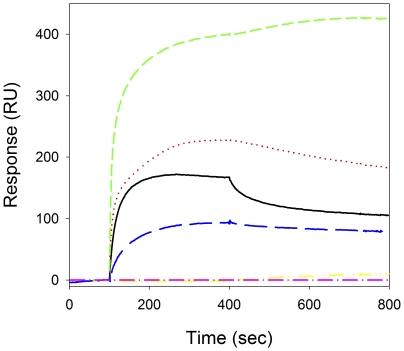
Screening of amine coupled Str enolase with different proteins. The F137L/E363G Str enolase coated chip was tested for binding with 500 nM of each of the following proteins: (from top to bottom) yeast enolase, Str enolase, dPgn, BSA and MBP.

A variety of label-free, real-time Surface Plasmon Resonance (SPR) experiments were performed. Both F137L/E363G enolase and plasminogen were coupled to SPR chips using amine coupling. Preliminary screening of the Str enolase coated chip showed that dPgn, Str enolase, fatty acid free bovine serum albumin (BSA) and yeast enolase would bind to the coated surface (Figure 6). Maltose binding protein (MBP) showed no significant binding to the Str enolase coated surface. The dPgn coated chip was also screened. Like the Str enolase chip, it bound dPgn, Str enolase, fatty acid free BSA and yeast enolase; MBP did not bind (data not shown).

**Figure 7:**
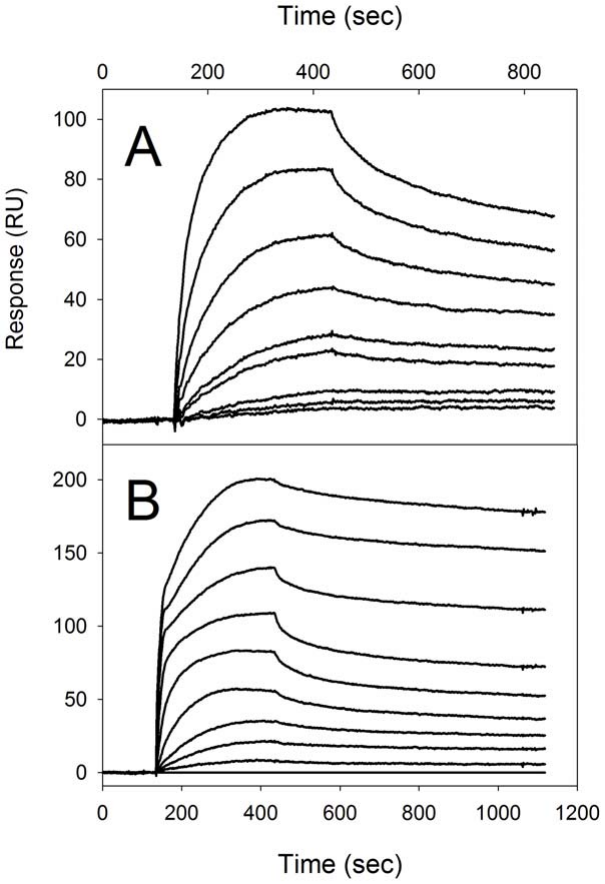
Titration of the amine coupled Str enolase with dPgn (A) and the amine coupled dPgn with Str enolase(B). 7A. In the top curve, the dPgn concentration was 500 nM. It was diluted 1∶1 in each successive descending curve. The bottom trace is the baseline. The reaction appears to be totally reversible. 7B. Titration of amine coupled dPgn with Str enolase The dPgn clearly binds enolase in a multiphase reaction. The initial phase is very rapid and does not appear to be reversible on the time scale of the experiment. The second phase appears to be reversible. In the top curve, the Str enolase concentration was 500 mM. It was diluted 1∶1 in each successive descending curve.

Next, a multi-cycle titration of the F137L/E363G coated chip was performed with soluble, native dPgn (Figure 7A). The association phase was monophasic. The dissociation phase indicated that the off rate was about 10% of the on rate. The steady-state amounts of dPgn bound indicated that the apparent dissociation constant for the complex is in the submicromolar range. It is important to note that coupling of Str enolase to the amine based chip was performed at pH 4.5 (see Table 2) where the electrostatic attraction between protein and chip was experimentally determined to be strongest.

The dPgn coated chip was titrated with F137L/E363G Str enolase (Figure 7B). The titration was significantly different from that of F137L/E363G Str enolase titrated with dPgn. The association phase was multi-phasic; the dissociation rate was very slow and dissociation did not appear to go to completion. Half saturation of all the association phases occurred at about 50 nM Str enolase but this is number has no real meaning since the on reaction is clearly multiphasic and the off reaction does not appear to go to completion.

We also performed SPR using Ni-NTA coated chips; in these experiments, the Str enolase was bound, at neutral pH, via a poly-histidine tag. Three histidine-tagged Str enolase variants were initially screened for their ability to bind to Ni^2+^-activated NTA sensors (Figure S1). The C-terminal His_10_-wild type Str enolase showed the strongest binding followed by N-terminal His_6_-F137L/E363G- Str enolase. N-terminal His_6_-wild type Str enolase did not bind to any significant extent. As expected, there was no capture of the His-tag free controls (i.e. dPgn and the wild type enolase with no tag).

**Figure 8:**
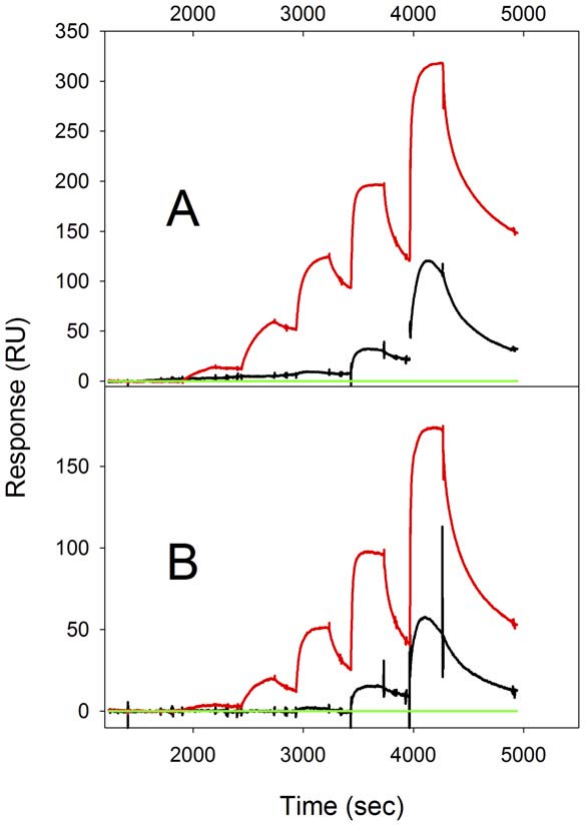
Binding of proteins to his-tagged Str enolase coupled to Ni-NTA chips. Figure 8A. Different proteins binding to Ni-NTA immobilized F137L/E363G Str enolase. The enolase was titrated with dPgn (red), wt-Str enolase with no tag (black) and MBP (green). Each set represents titration with 15 nM, 62 nM, 125 nM, 500 nM and 2000 nM protein followed by washout. At the lower concentrations of titrating protein there is some specificity for dPGN which disappears at higher concentrations. Figure 8B. The wt-Str enolase was immobilized on Ni-NTA via the carboxyterminal deca-his-tag. dPgn (red), wt-Str enolase with no tag (black) and MBP (green) were flowed over the immobilized protein at concentrations of 15 nM, 62 nM, 125 nM, 500 nM and 2000 nM. There is some specificity at the lower concentrations but not at the higher. The two titrations are very similar but differ in the total response and the response to low concentrations of titrant protein.

Figure 8A illustrates binding to the F137L/E363G coated chip when challenged with dPgn, MBP or His-tag-free enolase. BSA binding is not shown; it was similar to the tag-free enolase binding at all concentrations. At low concentrations of dPgn, there is significant binding to the immobilized enolase. Tag-free native enolase, at low concentrations, also binds to the immobilized enolase, but this is considerably less than the amount of dPgn that binds. At high concentrations of ligand, the immobilized Str enolase bound dPgn, tag-free enolase and BSA but not MBP. The maximum observed binding of dPgn to the immobilized enolase correlated well with the predicted binding maximum for a stoichiometry of dPgn∶enolase monomer = 1∶1 (see Methods).

Figure 8B illustrates binding to the chip when coated with C-terminal His_10_-tag wild type enolase and then challenged with dPgn, BSA, MBP or His-tag-free enolase. The same three proteins bind in approximately the same ratios as with the N-terminally tagged enolase. Native, octameric Str enolase should not, in our opinion, bind additional enolase. This can only mean that the enolases bound directly on the chip are, at least partially, not in their “native” state.

We have maintained up to this point that binding between the various partners occurs because the native structure of the proteins is disrupted by the conditions leading up to binding. Can we corroborate this idea with additional data? We used analytical ultracentrifugation to report on the quaternary structure of the protein, activity assays to report on the degree to which the active sites of Str enolase were intact, and circular dichroism to report on the secondary structure of the enolase.

### Enolases at low pH

When diluted into acetate buffer, pH 4.0 or 4.5, F137L/E363G enolase was inactive; in the AUC, there was a broad peak in between that expected for octamers and monomers. No other species were present. In other experiments, enolase precipitated within minutes of being diluted into this buffer. Our results at low pH were somewhat variable but it was clear that pH 4–4.5 treated F137L/E363G enolase lost activity and lost its octameric structure. Are the effects of this buffer treatment reversible? Enolase was incubated at pH 4.5 for one hour and dialyzed overnight against the buffer used in the SPR for the ligand binding. Approximately 60% of the initial protein was recovered in the soluble fraction of which 75% was monomeric and 19% octameric. Its specific activity was 28% of the native but it maintained 75% of the peptide bond CD signal. We conclude that exposure of the *S. pyogenes* enolase to pH 4.0 or 4.5 buffer leads to precipitation, dissociation and partial unfolding. These effects are not fully reversible. As shown by SPR (Fig. 7), fluorescence polarization (Table 2) and ITC (Fig. 3), acid treated protein binds dPgn.

### “Native” enolases at neutral pH in SPR binding buffer

This buffer (see Materials and Methods) does not contain Mg^2+^ but does contain EDTA. These conditions are known to destabilize other enolases [50], [51]. As shown in Table 3, the various constructs of his-tagged Str enolase are all partially dissociated in this buffer. There is a clear correlation between the amount of enolase captured by the NTA sensor chip and the percentage of the enolase that is monomeric in this buffer.

**Table 3:** Quaternary Structure of Enolase in SPR binding buffer as determined by AUC.

Enolase	% octameric	% monomeric
F137L/E363G	57	36
wild type, N-terminal tag	88	5
wild type, C-terminal tag	68	18

See Materials and Methods.

According to the X-ray structure of the *S. pneumoniae* enolase (1w6t.pdb, [44]) the histidine tags point into the center of the ring of subunits. The fact that the NTA sensor chip could bind F137L/E363G enolase and wild type enolase with a C-terminal histidine tag, but not the wild type enolase with an N-terminal tag suggests to us that the N-terminal tag of the octamer is not accessible for binding to the chip; the form of enolase binding to the Ni-NTA chip is most probably monomeric.

### Binding in the presence of phospholipid micelle surfaces

**Figure 9:**
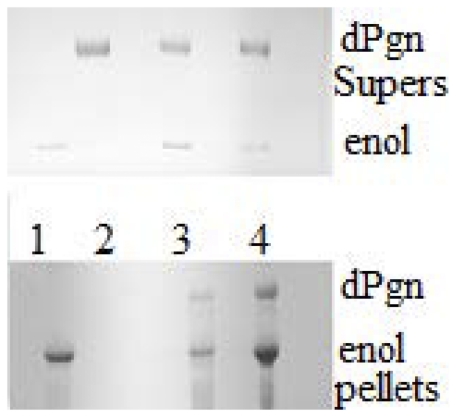
Enolase adsorbed to the surface of phospholipid micelles will bind dPgn. Azolectin (2 mg/mL) was suspended in 10 mM potassium phosphate, 100 mM NaCl, pH 7 and sonicated until clear. Str enolase, when present, was added to 300 nM; dPgn when present was 1000 nM. The first protein addition (Experiment 3: Str enolase, experiment 4, dPgn) was made to the azolectin micelles and the solution sonicated for another two minutes. The second protein addition was made was made and the solutions incubated at 4°C for three days (convenience timing). The samples were centrifuged at 20 000 rpm at 4°C for 2 hours. The phospholipids were extracted with isopropanol, chloroform and the remaining material dissolved in SDS sample buffer. The SDS-PAGE patterns of the pellets and supernates is shown. Lane 1 contained Str enolase only. The micelles pull down the enolase. Lane 2 contained dPgn only. The micelles do not pull down the dPgn. Lanes 3/4 contained Str enolase and dPgn. The micelles pull down the enolase which pulls down the dPgn.

In addition to SPR, we assessed surface-mediated binding of dPgn and Str enolase using liposome experiments. We adsorbed Str enolase to phosphatidyl choline (soybean) micelles and determined that, when centrifuged, plasminogen will cosediment with the micelles as shown in the gels of Figure 9. The gels were scanned and quantified. In both experiments, the majority of Str enolase (64% in experiment 3, 94% in experiment 4) sedimented with the micelles. The amount of dPgn, relative to the amount of Str enolase, that co-sedimented was 1∶1 to 1∶1.2. Cosedimentation of micelle and dPgn occurs only if enolase is also present; the micelles, by themselves, do not bind dPgn. The supernates and pellets were assayed for enolase enzymatic activity and the ability of Pgn to convert to plasmin. Str enolase in the supernate was active; it was also active in the pellet. dPgn in both supernate and pellet was slowly activated by trypsin. After 24 hours, about 50% of the dPgn in the pellet converted from glu-dPgn to lys-dPgn while the remaining 50% converted to plasmin.

## Discussion

We have posed the question: what is binding to what? We have approached our question by asking what are the conditions that promote the binding of Str enolase and plasminogen and what are the conditions that do not promote binding. The answers to these two questions have allowed us to sketch out a qualitative answer in terms of the overall thermodynamics of binding and the overall physiological importance of binding.

### A qualitative view of the thermodynamics of the binding

The native structures, plasminogen and Str enolase, are stable and show little tendency to unfold. For binding of a partner to occur, some degree of non-nativeness is necessary. For the reaction,
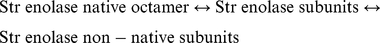
equilibrium favours the octamer as we isolate it and work with it in TME buffer. Our AUC experiments, however, indicate that octameric enolase does not retain its integrity under conditions such as the pH 4.5 treatments in our experiments, nor in the presence of SPR binding buffer. The addition of native plasminogen to native Str enolase does not perturb the equilibrium in such a way that we can detect it using the many techniques discussed here. ΔG for the overall reaction above is sufficiently positive and the energy barriers for the interconversions are sufficiently great that the addition of a potential binding partner cannot pull the equilibrium towards the non-native state necessary for binding. But, once the protein is converted to a non-native state, via a pH change or other perturbation, the energy barriers for proper refolding are sufficiently great that it does not occur on the time scale of our experiments. The resultant conformational changes in Str enolase favour its interaction with native plasminogen, but also with other proteins, such as BSA, Str enolase, and yeast enolase. Have we found any conditions in which our two principle proteins will bind to one another in a native state? The answer to the question is no. The closest we have come to maintaining totally native proteins that still show binding is in our SPR experiments using enolase bound to a Ni-NTA chip. Here the hexa-his tag is at the N-terminus of the enolase or at the C-terminus as a deca-his tag. For the hex-his, this protein will bind plasminogen but it will also bind albumin and more importantly Str enolase. The significance of this last point is that it must mean that the species contains significant amounts of non-octamers. Octamers show no tendency - that we can detect by AUC - to bind octamers. For the C-terminal deca-his tag, the Str enolase binds the same three proteins to about the same extent. In contrast to other studies [47], our data show that, in these experiments at least, C-terminal lysines are not required for binding. The two different his-tagged proteins do not differ in their binding capacity by a factor of more than three, even though the proteins must be in different orientations on the chip. We consider that for dPgn binding to Str enolase the data are clear. At least one of the partners must be non-native in order for binding to occur.

We are not able to define the specific forms that bind, but we can define the forms that do not bind. When both proteins are soluble, correctly and completely folded and, in the case of enolase, fully active, no binding occurs. Binding occurs in solution when the enolase has been subjected to the treatments used in SPR or ELISA experiments (Table 2, Figure 3). This enolase is a mixture of forms - monomeric and octameric, folded and unfolded, active and inactive; we do not know which form(s) bind plasminogen. The most we can say about binding during the SPR experiments that used amine coupling is that 1) very little, if any, octameric, active enolase was present and 2) binding was relatively non-specific, with Str enolase binding dPgn, BSA, yeast enolase and itself.

The binding of his-tagged Str enolase to Ni-NTA chips was used in SPR experiments in order to avoid the pH 4.5 treatment. The binding is more specific, with binding of dPgn being greater than that of more enolase or BSA. Based on the data in Table 3 and the relative amounts of enolase that could be immobilized, this protein is monomeric. The calculated stoichiometry of binding indicates that all of these monomers are capable of binding dPgn. CD spectra show that Str enolase in SPR buffer has normal secondary structure. However, the fact that monomeric Str enolase is inactive [52] means that there are some structural changes. We tentatively conclude, therefore, that the form of Str enolase that is capable of specific binding of dPgn is a folded monomer with an altered conformation. What the structure is of the enolase bound to cells is an open question at this time. We emphasize that, since bacteria which have enolase on their surface are found in many different environments, ranging from neutral to acidic, there may not be a unique answer to this question.

We cannot generalize these results to all instances of enolase-Pgn interactions, since, in other reports, information about the structure of the enolase under the various experimental conditions is lacking. Based on what is known about conditions that destabilize the quaternary structure of yeast and mammalian enolases [50], [51] and the Str enolase used in these studies, it is likely that the conditions used in SPR for coupling, for binding and for regeneration of the chip [36], [53] and conditions used in ELISA experiments would not maintain the native structure of most enolases. Our results with dPgn and Str enolase are similar to those reported for Factor H and C-reactive protein [53]. No binding between these proteins was observed when both were in solution nor when the C-reactive protein was immobilized under conditions that maintained its native structure. Binding was observed only under conditions that destabilized the structure of the C-reactive protein.

### Is there a physiological relevance to the binding of the two partners?

Are our results relevant to the proposed role of enolase during bacterial infection? To date, nothing is known about the structure of enolase when bound to cell surfaces, nor the mechanism by which this cytoplasmic protein arrives at the cell surface. Several groups [48], [54] have shown that isolated enolase will bind to bacterial cells. It has been proposed [55] that enolase is secreted, by an unknown mechanism, and then binds to the surface of the cells. Our experiments show that Str enolase binds to a phospholipid micelle and can then bind dPgn. At this point, we have no information about the structure of the enolase when bound to the micelles. Our results, both with the micelles and with the precipitating dPgn (Figures 4, 5 and 9), suggest that surfaces play a role in the enolase-Pgn interaction; perhaps the interaction of enolase with a surface produces a conformation of enolase capable of Pgn binding.

Str enolase and the other enolases found on cell surfaces will bind plasminogen but will also bind other proteins. A pathogen with surface enolase in the blood of a host is confronted with 0.002 mM plasminogen but also with ca. 0.500 mM albumin! When the number of enolase sites on the pathogen is large, there will be some plasminogen binding to the pathogen's enolase; how many plasminogens vs albumins will be determined by the relative binding coefficients and concentrations. The bound plasminogen could, when activated to plasmin, promote the dissemination of the infectious particles. More importantly, bound surface albumin or Pgn [56] might very well disguise the pathogen thereby allowing it to escape detection by the host's immune system. What is clear is that the surface enolase can bind a significant number of different species and these species likely determine the overall physiological outcome of the binding. A final complication in evaluating the physiological importance of this interaction is that it is also observed in bacteria viewed as “friendly”. Commensal microorganisms that have been shown to have surface enolases that bind plasminogen include oral streptococci [13], bifidobacteria [18] and lactobacillus [57]. Does the enolase on the surface of these bacteria have a physiological role or is its presence purely adventitious? Our rhetorical question does not have an answer at this time.

## Materials and Methods

### Ethics Statement

The experimental protocol was approved by the Animal Ethics Committee of the McGill University Health Centre Research Institute and the McGill University Animal Ethics Committee, in accordance with Canadian regulations for experimentation in animals. Dogs were maintained within the Large Animal Facilities of the MUHC-RI for the overall duration of experiments. The Unit is certified for Good Animal Practice Care by the Canadian Council on Animal Care. The animal work was carried out under protocol number 5717.

### Protein preparation and purification

The Str enolase gene, both wild type and the F137L/E363G mutant, were maintained in the pET-14b plasmid. In order to have a protein with a C-terminal histidine tag, the gene for the wild type protein was transferred into pET-52b(+). Mutagenesis, expression, purification using a Ni-NTA column under non-denaturing conditions and assays for enzymatic activity have been described [52]. The cleavage of the C-terminal his-tag to yield the Str enolase-WT was performed as follows: Thrombin (GE Healthcare) was suspended in 140 mM NaCl, 10 mM Na_2_HPO_4_, 1.8 mM KH_2_PO_4_, 2.7 mM KCl. Purified enolase was incubated with thrombin (1 unit thrombin per 0.001 mg protein) for 8 hours at 15°C. The reaction was stopped by dialysis against the same buffer; the cleaved mixture was contained within a membrane with a 50 000 molecular weight cutoff through which the thrombin could pass. The high molecular weight Str enolase without the his-tag was passed through a 1 mL Ni-NTA column. The fraction that did not bind, tag-free Str enolase, was dialyzed vs TME.

The preparation of dPgn has been described [58]. Our standard assay for dPgn activation to plasmin consists of incubating dPgn with trypsin (40 nM), monitoring enzymatic activity using Spectrozyme and monitoring the change in gel pattern as the dPgn converts first to lys-dPgn and then to plasmin.

All five proteins were estimated to be >95% pure by SDS-PAGE. The standard buffers used were:

1. TME, 50 mM Tris, 1 mM Mg(OAc)_2_, 0.1 mM EDTA, pH 7.4.
2. Phosphate buffer, 5 mM KH_2_PO_4_, 5 mM K_2_HPO_4_, 100 mM NaCl, pH 6.7.
3. SPR precoupling buffer, 20 mM sodium acetate pH 4 to pH 5.
4. SPR binding buffer (amine coupled chips), 10 mM HEPES, 150 mM NaCl, 1.4 mM EDTA, pH 7.4.

All chemicals were of the highest quality available and were purchased from Fluka Chemical Co., Sigma Chemical Co., Fermentas, Stratagene, or Pharmacia; Spectrozyme was from American Diagnostica.

*Dynamic light scattering* was performed on a Wyatt Technology Corp. DynaPro instrument equipped with temperature control. Scattering was measured at 20°C in a 0.025 mL cuvet. Buffers and samples were centrifuged for 5 minutes at 13,000 RPM in a benchtop microfuge. A 0.040 mL aliquot was taken from the top of the centrifugate and placed in the cuvet with a Hamilton syringe. Water and buffer blanks contributed about 3E4 counts per second. Samples containing protein contained a sufficient concentration to give about 2E6 counts per second. Data were collected for 10 seconds per acquisition; 100 acquistions were collected. At least 95% of the acquisitions were of acceptable quality. Data were evaluated using the Wyatt software. Occasionally we checked the results qualitatively using Sedfit.

*Sedimentation velocity experiments* were performed in a Beckman XL-I analytical ultracentrifuge at 32 000 rpm at 20°C and monitored at either 280 and or 230 nm. The partial specific volume of the proteins, solvent density and viscosity were calculated by SEDNTERP, version 1.07 (D.B.Hayes, T. Laue and J. Philo, available at www.bbri.org/RASMB/rasmb.html) and the results were analyzed with the Sedfit program [59], [60].

*Fluorescence polarization* was performed on a Cary Eclipse fluorometer equipped with an automatic polarizer attachment. The function of the polarizers was verified using a light scatterer (dilute milk) as standard.

*Isothermal titration calorimetry* was performed using a Microcal VP-ITC calorimeter thermostated at close to 20°C. The protein solutions were dialyzed against three buffer changes. The data were analyzed using Origen software provided by Microcal. Baselines required adjusting in all cases and were treated using the same software. Rigorous thermodynamic parameters were not calculated because the concentrations of the various species in solution, after the different pH treatments, could not be determined.

*Circular dichroism (CD)* spectra were measured at 15°C with a Jasco J-810 spectropolarimeter. For each protein sample its buffer solution was scanned to obtain the baseline. Samples were scanned 5 times with the response time of 1 second and bandwidth of 1 nm from 260 to 200 nm at a scan rate of 20 nm/min. The Spectra Manager program (Jasco) was used to subtract the baseline and smooth the spectra. Spectra of the native enolases were assessed in TME while the pH 4.5 treated proteins were in SPR binding buffer.

*“Pull down” reactions* were of two types: (1) DTT induced precipitation and the course of the reaction followed at 280 nm have been described [49] and (2) adsorption of Str enolase and dPgn to phosphatidyl choline micelles. In the latter case, azolectin (2 mg/mL) was suspended in 10 mM potassium phosphate, 100 mM NaCl, pH 6.7 and sonicated until clear. Str enolase, when present, was 300 nM; dPgn, when present, was 1000 nM. The first protein addition was made to the azolectin micelles and the solution sonicated for another two minutes. The second protein addition was made and the solutions incubated at 4°C for three days (convenience timing). The samples were centrifuged at 20 000 rpm at 4°C for 2 hours. The phospholipids were extracted with isopropanol/chloroform and the remaining material dissolved in SDS sample buffer. The samples were analyzed by SDS-PAGE. The developed gels were scanned and quantified with ImageJ, available from NIH.

*Surface plasmon resonance* was performed on a BIACORE 3000 instrument (GE Healthcare Bio-Sciences AB, Upsala, Sweden). Amine-coupled experiments were performed on CM4 sensor chips at 25°C. Alternatively, Ni-NTA chips were used to assay binding by the hexa-his or deca-his tagged Str enolases. Initially, F137L/E363G Str enolase (5 µg/mL in 20 mM sodium acetate pH 4.5) was directly immobilized to CM4 sensor chips using the Biacore Amine Coupling Kit (∼260 Resonance Units (RU) final); corresponding reference surfaces were prepared in the absence of enolase. Purified dPgn, bovine serum albumin (BSA), Maltose Binding Protein (MBP), yeast enolase, or Str enolase were injected (500 nM each) over reference and randomly-oriented Str enolase surfaces (30 µL/min×5 min association+5 min dissociation). Between sample injections, sensor chip surfaces were regenerated at 50 µL/min using two 30-second pulses of running buffer containing 1 M NaCl, 10 mM NaOH, and 0.5% (v/v) Empigen) and Pierce Gentle Elution buffer containing 0.5% (v/v) Empigen. dPgn was then titrated (0–500 nM; 2-fold dilution series) in a similar manner over Str enolase-immobilized surfaces (0.030 mL/min×5 min association+10 min dissociation). In reciprocal experiments, Str enolase was titrated (0–500 nM; 2-fold dilution series) over reference and amine-coupled dPgn surfaces (0.005 mg/mL in 20 mM sodium acetate pH 4.5; 340 RU immobilized to CM4 sensors).

To complement the direct amine-coupled experiments, Str enolase variants were indirectly captured to NTA-coated sensor chips to (i) avoid the acidic immobilization conditions and (ii) create homogenous, oriented surfaces. Initially, the four different Str enolase variants (each diluted to 100 nM in the presence of 250 mM NaCl and 10 mM imidazole) were injected over activated NTA surfaces to compare their capture efficiencies under identical assay conditions. To ensure that each variant was stably bound, post-capture surfaces were washed at 0.05 mL/min using two 30-second pulses of 10 mM HEPES, 500 mM NaCl, 10 mM Imidazole, and 0.05% (v/v) Empigen, followed by 10 mM HEPES, 150 mM NaCl, 0.05 mM EDTA and 0.005% (v/v) Empigen. Surfaces were considered stable if there was no significant dissociation over 180 sec post-washing. Between sample injections, sensor chip surfaces were regenerated at 0.050 mL/min using two 1-min pulses of Pierce Gentle Elution solution containing 0.1% (v/v) Empigen followed by 200 mM imidazole, and finally 350 mM EDTA. Subsequently, single-cycle titrations were performed using NTA-captured Str enolase surfaces: N-terminal His-tagged F137L/E363G and C-terminal His-tagged wt Str enolase (∼150 RU of each variant captured per cycle). dPgn, tag-free Str enolase, MBP, and BSA were injected (0–2000 nM; 4-fold dilution series) over NTA reference and Str enolase-captured surfaces (0.010 mL/min×5 min association+1–10 min dissociation); between sample cycles, surfaces were regenerated as noted above.

All SPR data were doubled-referenced [61] and is representative of duplicate injections acquired from two independent trials. For the amine-coupled experiments, a buffer blank was injected at the start of each multi-cycle titration, the highest titrant concentration second, and serial dilutions followed (from the lowest to the highest concentration). Comparing responses between the two highest titrant injections verified consistent immobilized surface activity throughout each assay. Steady-state binding responses (RU at end of association phase) were plotted as a function of titrant concentration; apparent equilibrium dissociation constants (*K*_D_) were determined by global fitting of the data to a “steady-state affinity” model (BIAevaluation v4.1 software). For the NTA experiments, the single-cycle titrations were fit to a “1∶1 Titration” model in the BIAevaluation software [62]. Theoretical binding maxima were predicted using the following equation: R_max_ = (MW_A_/MW_L_) (R_L_) (n); ‘R_max_’ is the theoretical maximal binding response (RU) at saturating titrant concentration, ‘MW_A_’ is the molecular weight of the titrant injected in solution, ‘MW_L_’ is the molecular weight of the immobilized biomolecule, ‘R_L_’ is the amount (RU) of biomolecule immobilized, ‘n’ is the predicted binding stoichiometry (e.g. 1∶1).

## Supporting Information

Figure S1 Screening for the capture of protein on a Ni-NTA sensor chip. The proteins flowed over the chip were dPgn (solid), N-terminal Hexa-histidine F137L/E363G (dots), Hexa-histidine wt (dash) and Deca-histidine wt (dash dot). Additionally, the chip was challenged with NaCl and the deca-histidine cleaved enolase; these, like the dPgn, did not stick to any significant extent. Prior to second 400, the chip was washed with EDTA and NiSO_4_. Ligand capture occurred during phase III. Phase IV was a high salt wash and phase V was a buffer wash.
(TIF)
